# Mobilizing grievances in the internet age: The case of national online petitioning in South Korea, 2017–2022

**DOI:** 10.1371/journal.pone.0302373

**Published:** 2024-05-16

**Authors:** Kayoon Kim, Chan S. Suh

**Affiliations:** 1 Quantitative Data Science Methods, The University of Tübingen, Tübingen, Germany; 2 Department of Sociology, Chung-Ang University, Seoul, Republic of Korea; Alzahra University, ISLAMIC REPUBLIC OF IRAN

## Abstract

Which kinds of grievances garner support from the public on online platforms? Focusing on national online petitioning, one of the forms of direct democracy in contemporary politics, we examine the content and characteristics of petitions that succeeded in attracting public attention and support. Using our comprehensive data on online petitions that were submitted to the executive office between 2017 and 2022 in South Korea, our analysis yields three important findings. First, a mix of post-materialist topics such as human rights and gender equality and materialist topics such as safety and environment turn out to be salient among petitions that meet the signature threshold. Second, online petitions the contents of which reveal either moral emotions or Confucian attitudes are more likely to gain public support compared to others. Third, keywords that are related to moral claims asking for the apprehension of perpetrators on behalf of victims, such as ‘victim,’ ‘perpetrator,’ ‘kid,’ and ‘punishment,’ appear most frequently inside the petitions that cross the signature threshold. Such findings provide implications for understanding both the potentials and limitations of national online petitioning in contemporary democracies.

## Introduction

In stable democracies, institutional arrangement has been devised to guarantee citizen participation in politics. Under a democratic system, not only have the checks and balances been implemented against the administrative power by legislative and judicial bodies, but opportunities have been granted to actors in the civil society to engage in political decision-making processes [1,2]. A variety of grievances that different constituencies raise could also transform into collective or individual claims. Social movement activism such as rallies, sit-ins, and boycotts has been embraced by the public as a legitimate part of democracies [3,4], and legal mobilization—using litigation tactics to express grievances and demands in lawsuits—has also been adopted to challenge institutional actors through independent judicial processes [5–8]. Submitting petitions to the government has also been identified as a well-established repertoire among disadvantaged citizens to engage in institutional politics [9].

The rise of information and communication technology (ICT) has further enhanced the quality of representative democracy. Although new problems such as increasing political polarization and extremism have emerged in the backdrop of digital transformation [10,11], the Internet has obviously provided an alternative public space within which individuals from diverse backgrounds could more easily engage in online activism using digital repertoires [12,13]. Social media and digital discussion forums have facilitated the communication of collective and individual grievances among citizens [14–17]. From the Arab Spring in North Africa and the Middle East to the anti-austerity movement across Europe, the Occupy Wall Street movement in the United States, and the Umbrella Movement in Hong Kong, protests could rapidly spread with the assistance of such online platforms. Also, national online petitioning websites have emerged as a form of nascent, low-cost political participation [18,19]. Online petitioning has been praised for their potential to narrow the gap between institutional political processes and citizens’ grievances [20].

Submitting petitions to the government itself has been a traditional repertoire; in Great Britain, where the Parliament enhanced its power in the early modern era, the submission of petitions to the Parliament has been one of the conventional repertoires for workers to raise their voices [21]. With the rise of the Internet, however, sending online petitions to the government has become an increasingly popular repertoire for citizens to make individual claims [13]. Governments have also launch national online petitioning platforms upon which individuals could share their grievances and potentially get formal responses from the government. A prominent example includes the U.K. Parliament petitions website, which started its operation in 2006 and was formalized as a perpetual part of the government in 2015. In 2011, the U.S. White House also created a platform called “We The People,” where people could generate and sign petitions. Benchmarking these efforts, governments in non-Western countries—including the Blue House in South Korea—have also adopted similar online petitioning platforms.

Corresponding to the inter-governmental diffusion of online petitioning platforms, prior research has begun to examine the nature of the online petitions that are filed through these platforms. Some studies have revealed the basic characteristics of petitions that are filed by the public [22,23], while others have exclusively focused on the dynamics by which petitions become successful [24]. Despite the increasing interest in online petitioning, however, research has predominantly focused on Western countries. This is a serious limitation, given that the political and cultural dynamics of online petitions differ between Western and non-Western countries. More specifically, the subject and content of grievances that are filed and then supported by the public within online platforms may be different in non-Western countries. In this regard, our study on the case of South Korea can provide a unique opportunity to identify the process by which grievances garner public support on online platforms and, thereafter, evolve into political agenda.

Among non-Western countries, we choose South Korea as our case for three reasons. First, South Korea has not only experienced rapid industrialization but also developed one of the fastest Internet infrastructure services. As the country successfully went through digital transformation, citizens have utilized online platforms as their primary sources of communication. Second, South Korea has developed a vibrant civil society following a successful democratic transition in 1987 [8,25]. In the face of such rapid social change, diverse social and political constituencies have made a variety of collective claims with the use of various online and offline movement repertoires. Finally, the online petitioning platform run by the Blue House in South Korea is a successful example of the public vigorously sharing their concerns and claims via the Internet [26]. The total number of online petitions that are made on this platform during the five-year period from 2017 to 2022 reached 1,100,000, with petitions that received 100 or more signatures exceeding 34,000. Some of the petitions also successfully became prominent agendas in both the civil and political societies in South Korea.

While South Korea has experienced rapid modernization through both industrialization and democratization, it has also been influenced by the historical and cultural legacy of Confucianism. Therefore, this non-Western case provides a unique opportunity to see the coexistence of modern and traditional attitudes of citizens who adopt direct democratic repertoires. While prior efforts have been made to examine the characteristics of online petitions that lead to a high volume of signatures in the case of South Korea [26,27], we adopt an inclusive data collection strategy to cover the entire five-year period from 2017 to 2022, when the Blue House petition platform was active. Our comprehensive data include the entire sum of online petitions that are listed on the website. Using this data, we apply regression models to examine which characteristics in those petitions increase the likelihood of receiving significant support from the public. Then, we employ both sentiment and content analyses to measure the internal characteristics of these online petitions that received public support. Our findings suggest that the most popular topics include a mix of post-materialist ones such as human rights, gender equality, and companion animals and materialist ones including safety and environment. Also, petitions that reveal negative emotions or Confucian attitudes, and petitions asking for the apprehension of perpetrators on behalf of victims are more likely to garner public support compared to others. The implications of these findings for the potentials and limitations of online petitioning are discussed in the conclusion.

## Data and methodology

We collect a unique, comprehensive dataset on online petitions that were submitted to the executive office from August 19, 2017, to May 9, 2022, under the Moon Jae-In Administration in South Korea. We use web crawling techniques to capture basic information from both the National Petition to the Presidential Office website and the Presidential Archives of Korea website. The website contains online petitions that received a minimum of 100 or more signatures. From these websites, we use an open-source scraper in crawling information (https://github.com/lovit/petitions_scraper). Web crawling is conducted using Python, and 45,766 petitions are stored in the MySQL database using the pymysql module. After collecting data, we adopt a two-stage analysis to first examine the petition-related factors leading to a great deal of public support using logistic regression analysis and, second, identify keywords that are used in the petitions that receive support with the application of natural language processing.

**Fig 1:**
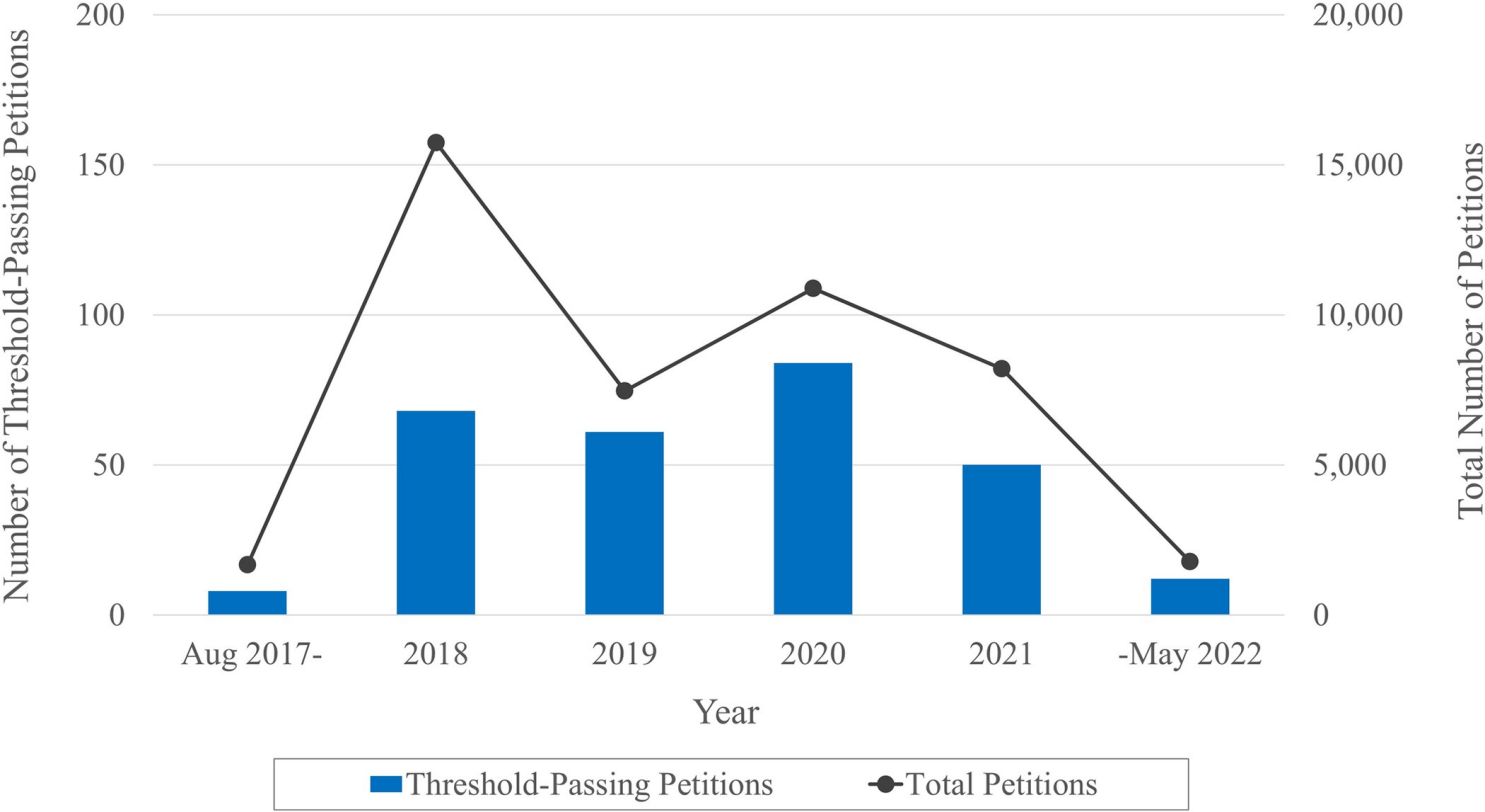
The number of petitions filed and passed over time: 2017–2022.

In the regression models, we examine the petition-related factors that increase the odds of receiving 200,000 or more signatures from the public—the threshold that is necessary to seek a response from the executive office. Once this threshold is reached, the petition also receives attention from the media and possibly becomes part of the social agenda. There are 283 petitions out of 45,766 that crossed the signature threshold in the given period. Fig 1 demonstrates the number of petitions that are filed and then passed across the signature threshold over time from August 2017 to May 2022. The beginning and final years of 2017 and 2022 mark lower numbers of petitions filed and passed compared to others since data is truncated before August 19, 2017, and after May 9, 2022. Other than the year 2018 when there were public attention to this new national petition system lead to higher number of petitions filed to the system, there is no obvious fluctuation in the numbers of petitions that are filed and passed over time.

There are three sets of independent variables that are used to explain the passage of the signature threshold in a regression setting. Firstly, we collect information on the subject category for each petition to see which subject categories are more likely to receive public support than others. A list of 17 subject categories is provided by the Blue House online petitioning website, and the list has not changed since the website was launched in 2017. Petitioners were able to choose which subject categories their petitions fall into, and thus, each petition is assigned a particular subject among 17 subject categories.

From the list, 17 binary variables are generated to examine the effect of each subject on receiving public support. The subject categories include (1) childcare and education, (2) companion animals, (3) culture, arts, sports, and media, (4) economic democratization, (5) economic growth engine, (6) employment, (7) foreign affairs and unification, (8) future, (9) human rights and gender equality, (10) low fertility and population aging, (11) political reform, (12) public administration, (13) public health and welfare, (14) rural (agricultural/fishing/mountain communities), (15) safety and environment, (16) transport, architecture, and land, and (17) others. Among the 17 categories, the “other” category necessitates further examination; Petitions that fall into the “other” category do not show any obvious pattern in their content as this category was selected when petitioners did not pay enough attention to find the exact category for their petitions. We use this category as the reference group for our regression analysis as a random mix of subjects is included in this category. Petitions that received the most signatures from the public within the “other” category include a mix of political and social topics such as “We support for President Moon Jae-In (1,504,597 signatures)”, “Impose a proper punishment on teenagers who killed others with a stolen rent car (1,007,040 signatures)”, “Asking for an extension of investigation period for (the sexual abuse of) late Jang Ja-yeon (738.566 signatures)”, and others.

Secondly, we conduct sentiment analysis to examine which types of sentiments are more likely to garner public interest and support than others. Sentiment analysis is a computational method used to assess people’s opinions, attitudes, and emotions toward an entity [28]. This analysis is considered a natural processing language task to estimate one’s sentiments about an entity. In order to measure the probability of revealing various types of sentiments from online petitions, we utilize KOTE (Korean Online That-gul Emotions), a fine-tuned language model based on a pre-training of KcELECTRA (Korean comments ELECTRA) using pytorch, pytorch-lightning, and transformers, with online comments written in Korean [29,30]. In KOTE, online comments extracted from 12 different domains are used to train data for fine-tuning. We rely on this KcELECTRA-based language model for measuring sentiments because of its ability to analyze informal texts that are not as refined as news articles or books. Using KOTE, we are able to label 44 different types of sentiments from the content of online petitions—from positive sentiments, such as feeling grateful, touched, favorable, or respectful, to negative sentiments, such as showing complaint, disappointment, anger and loath, sorrow, or doubt.

The third set of independent variables aims to assess the influence of four types of attitudes toward the likelihood of receiving public support that are revealed in the petition. In addition to sentiment analysis, we identified different types of attitude bases on our own own keyword analysis. After identifying a list of specific keywords as falling into the categories of an anti-immigrant attitude, appeal to people’s emotions, appeal to higher authority, and a family-oriented attitude, we sort out candidates of online petitions that may or may not reveal a particular attitude or an appeal when one of the keywords appears in its content. Keywords that we use to find petitions that may potentially fall into attitude/appeal categories include “refugees (난민)”, “Chinese (중국인)”, “immigrant (이민자)”, “Korean Chinese (조선족)”, “foreigner (외국인)”, and “foreign worker (외노자)” for an anti-immigrant attitude, “poor (불쌍)”, “suffering (고통)”, “pain (아픔)”, “excessively (너무나)”, and “desperately (간곡히/간곡하게)” as to one’s appeal to emotions, “subjects (백성)”, “servant (소인)”, “king (왕)”, “Mr. President (대통령님)”, “Your Majesty (폐하)”, “Your Honor (각하)”, “in consideration of (헤아리)”, and various expressions related to “beg you (해주세요/하시어/옵소서)” for an appeal to higher authority, and “brother (형)”, “my family (내 가족)”, “family (가족)”, “wife (집사람)”, “mother/mom (어머니/어머님/엄마)”, “father/dad (아버지/아버님/아빠)”, “daughter (딸)”, “a child (아이)”, “our children (자녀)”, “son (아들)”, “parent (부모님)”, “nephew (조카)”, “baby (애기/아기)”, and “husband and wife (부부)” as to family-oriented attitude. We manually read all the petitions that include these keywords and discern whether each petition reveals an anti-immigrant attitude, appeal to people’s emotions, appeal to higher authority, and a family-oriented attitude. As a result, four binary variables are generated where the value is 1 when either an anti-immigrant attitude, appeal to people’s emotions, appeal to higher authority, or a family-oriented attitude is revealed, and 0 otherwise. Each category is not mutually exclusive such that a single petition may fall into more than one attitude/appeal categories.

Finally, two additional control variables are included: a continuous variable that measures the actual length of a particular petition and a binary variable measuring the existence or non-existence of a URL (Uniform Resource Locator) reference for the given petition. As to the URL variable, a URL may be added in petitions to provide additional explanation on the significance of the petition in a separate webpage. While submitting online petitions involves low cost for petitioners [13], providing a URL within a petition is an additional work for a petitioner in preparing a petition. Therefore, we expect that petitions that are fully prepared with a URL are more likely to receive signatures from the public.

We are interested in examining which petitions receive significant public support. Using the variable on the number of signatures that a petition receives, we primarily focus on the likelihood that a petition meets the 200,000-signature threshold. We focus on petitions that pass the threshold requirement since civil society actors strategically mobilized around petitions to make them pass their signature threshold. While most petitions only attract a handful of supporters, those few successful petitions show which issues have been the major issues that have received considerable public support from the civil society. The official responses that these petitions receive from the executive office also lead to additional attention and support from the public.

Since the dependent variable is a binary one, we employ a series of binomial logistic regression models to test the effects of the three sets of independent variables [31]. The coefficients in the models estimate the effect of an independent variable on the log odds of passing the signature threshold, controlling for other variables. The stepwise regression technique is also adopted for the first and second sets of independent variables because of the large number of variables. Stepwise regression is the step-by-step iterative generation of a final regression model that identifies a parsimonious list of statistically significant independent variables.

In addition to meeting the 200,000-signature threshold, we assume that the number of signatures that a petition receives can provide additional lens to measure the level of support from the public. Accordingly, we add another table that basically replicate our regression models with a new dependent variable—the number of signatures for each petition. Due to the continuous nature of the dependent variable, we use ordinary least square regression models.

Following the regression analyses, we use natural language processing to analyze the actual content of the petitions that meet the threshold. In this second stage of analysis, our purpose is to identify keywords that are used in those online petitions. Natural language processing, a method used to extract data from a large body of human language and to analyze the meaning, intent, and sentiment of the given language, consists of three different processes: tokenization, cleaning, and token normalization. In the word-tokenization process, the given corpus of language is divided into tokens. Due to the characteristics of the Korean language, texts are tokenized in the unit of morphemes [32]. In the cleaning process, tokens with no meaning, such as particles and predicates, as well as signs and emoticons, are removed. Thereafter, we go through the normalization process to combine words that have similar meanings. In analyzing the normalized data, we use both graphs and a word cloud, a text-based visual representation of a series of tags [33], to efficiently visualize the keywords used in the petitions.

## Results

Which type of concerns and complaints receives greater support from the public on the national online petitioning platform in South Korea? To begin with, we start with a descriptive figure that shows the number of online petitions that were listed on the petitioning platform, classified by the petition subject.

**Fig 2:**
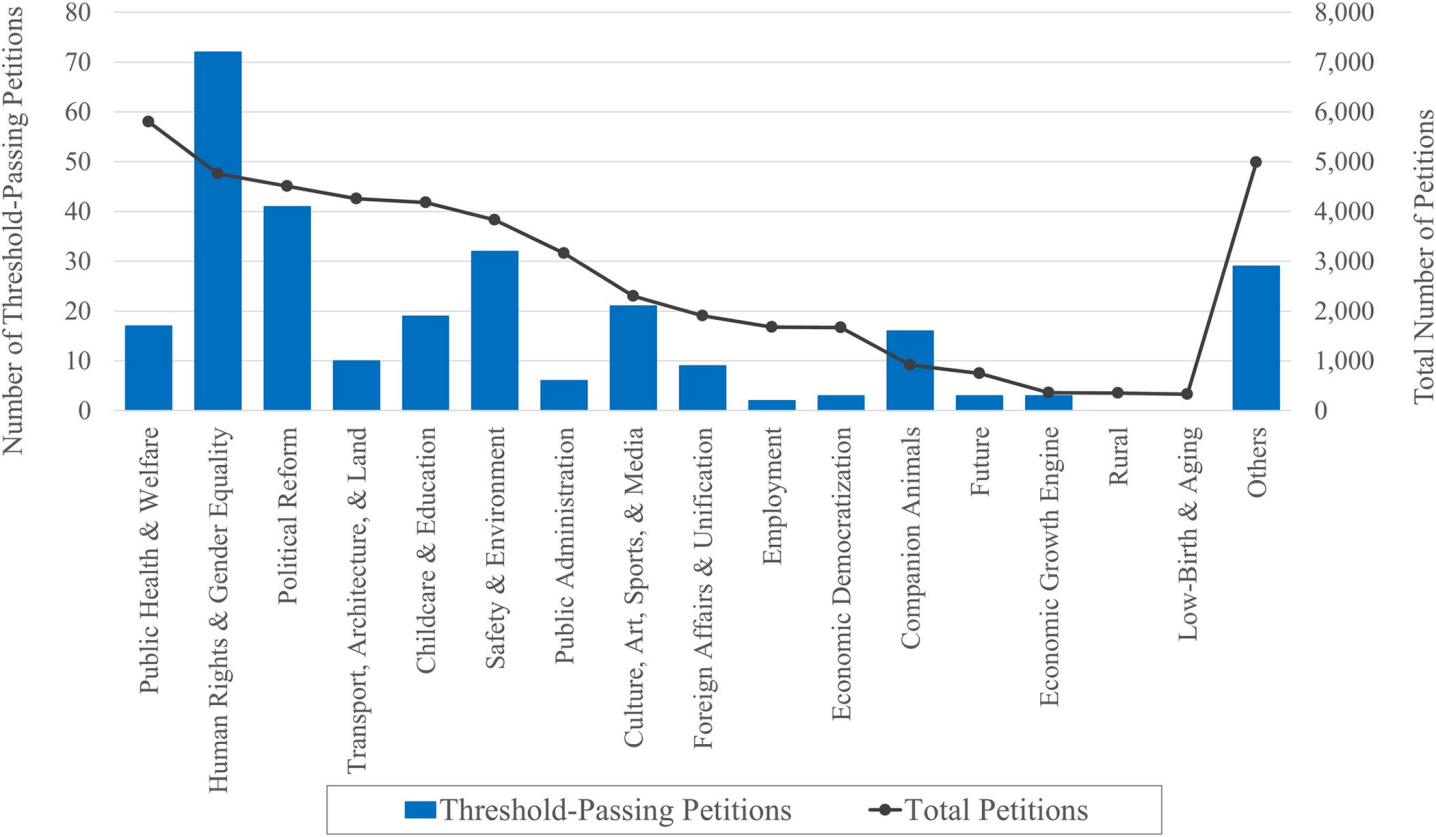
The frequency of keywords in online petitions that meet the signature threshold.

There were 45,766 petitions that were shared on the online platform and 283 petitions that received 200,000 or more signatures from the public—the threshold necessary to seek a response from the executive office. Looking into the number of petitions filed by subject, Fig 2 shows that the ‘public health and welfare’ subject had the highest number of petitions listed on the platform (5,805 petitions, 12.7%), followed by the subjects ‘human rights and gender equality’ (4,761 petitions, 10.4%), ‘political reform’ (4,506 petitions, 9.8%), ‘transport, architecture, and land’ (4,257 petitions, 9.3%), and ‘childcare and education’ (4,184 petitions, 9.1%).

In comparison to the total number of petitions shared on the website, the subjects that drew attention and received support were slightly different. Among 283 petitions that crossed the threshold, 25.4% (72 petitions) were in the ‘human rights and gender equality’ subject category, followed by the subjects ‘political reform’ (41 petitions, 14.5%), ‘safety and environment’ (32 petitions, 11.3%), ‘culture, art, sports, and media’ (21 petitions, 7.4%), and ‘childcare and education’ (19 petitions, 6.7%). On the other hand, the subjects ‘public health and welfare’ and ‘transport, architecture, and land’ were less likely to receive a great deal of public support. Also, none of the petitions that passed the signature threshold fell into the subjects ‘rural (i.e., agricultural/fishing/mountain communities)’ and ‘low fertility/population aging,’ showing relatively less interest from the public in those subjects.

Moving on to the regression analysis, we first investigate which petition-related factors increase the likelihood of crossing the threshold of 200,000 signatures from the public. Specifically, we test which subjects of interest, as well as which emotional and content categories, are associated with the likelihood of garnering public support. As to the subject and sentiment categories where a large number of variables are generated from the content analysis, we first include all potential variables and then employ a stepwise regression analysis to identify a parsimonious list of independent variables (*p* < 0.001).

As shown in Table 1, Model 1 suggests that petitions that fall into the following five subject categories turn out to be positively and significantly related to meeting the signature threshold compared to others: (1) companion animals, (2) culture, arts, sports, & media, (3) human rights & gender equality, (4) political reform, and (5) safety & environment. In other words, petitions are more likely to receive great public support if their subjects fall into those five categories. It is noteworthy that four out of five categories are typically known as post-materialist topics—or issues referring to the need for political participation, care for the environment, an emphasis on creativity, and the enhancement of the quality of life [34–37]. At the same time, however, materialist topics related to safety/environment are also significantly related to receiving public attention and support in South Korea.

**Table 1:** Logistic regression models on the determinants of petitions crossing the threshold.

	Model 1	Model 2	Model 3	Model 4
*Subject Categories*:				
Companion Animals	1.64*** (.27)			1.06*** (.31)
Culture, Arts, Sports, & Media	.98*** (.24)			.89*** (.26)
Human Rights & Gender Equality	1.50*** (.16)			.68*** (.19)
Political Reform	.98*** (.19)			.63** (.21)
Safety & Environment	.89*** (.20)			.84*** (.21)
*Sentiment Categories*:				
Guilt		7.98*** (1.37)		6.14*** (1.67)
Loathing & Hate		2.49*** (.24)		2.22*** (.27)
Seriousness		1.64*** (.30)		1.51*** (.31)
Tiresomeness		-5.77*** (.94)		-5.36*** (.97)
*Attitude Categories*:				
Anti-Immigrant Attitude			.08 (.51)	.46 (.51)
Appeal to Emotion			.08* (.04)	.09* (.04)
Appeal to Authority			.02** (.01)	.02** (.01)
Family Orientation			.01* (.00)	.01** (.00)
*Controls*:				
Url Existence				.60** (.22)
Content Length				-.00 (.00)
Constant	-5.67*** (.10)	-6.58*** (.23)	-5.19*** (.07)	-6.95*** (.25)
N	45,766	45,703	45,766	45,703

Note

***p < .001

**p < .01

*p < .05 (two-tailed).

Next, Model 2 shows that, among 44 sentiment categories, petitions that involve sentiments such as (1) guilt, (2) loathing and hate, and (3) seriousness are positively and significantly associated with petitions crossing the threshold compared to others. On the other hand, petitions whose content includes a tiresomeness sentiment are significantly less likely to pass the threshold. Emotions, such as guilt, seriousness, and loathing and hate towards the wrongdoers, are related to one’s moral judgment, suggesting that moral emotions are necessary for increasing the likelihood of meeting the signature threshold.

Model 3 tests the influence of the four types of attitudes that we identify based on our own coding schema, and three attitude categories, namely appeal to emotion, appeal to authority, and family orientation, turn out to be positively and significantly associated with petitions passing the threshold compared to others. In addition to appeal to emotion, the attitude category that is relevant to our earlier findings, shown in Model 2, that moral emotions are critical, both appeals to authority and family orientation fall into the category of Confucian attitudes, which are persistent in South Korea. On the other hand, anti-immigrant attitudes do not have a significant influence on gaining support from the public.

Finally, Model 4 is our saturated model that includes all independent and control variables, and the results corroborate our earlier findings; the five subjects that are related to post-materialist values, the three emotional categories related to moral emotions, and the attitude categories that are relevant to Confucianism are positively associated with the odds of meeting the threshold of 200,000 signatures with statistical significance.

In addition to examining the petition-related determinants of the likelihood of passing the 200,000-signature threshold, we explore the factors that increase the number of signatures received by the public. As Table 2 suggests, the results corroborate our findings in Table 1 that petitions addressing post-materialist topics and revealing moral emotions or Confucian attitudes are more likely to receive public support than others.

**Table 2:** OLS regression models on the determinants of petitions receiving signatures.

	Model 1	Model 2	Model 3	Model 4
*Subject Categories*:				
Companion Animals	6629.84*** (1165.13)			3518.37** (1141.70)
Human Rights & Gender Equality	5114.94*** (540.59)			2293.58*** (582.58)
Political Reform	3383.72*** (553.73)			1623.66** (549.46)
Safety & Environment	3036.34*** (594.71)			1987.25*** (566.71)
*Sentiment Categories*:				
Guilt		44514.89*** (9877.30)		5909.82*** (862.54)
Seriousness		6398.73*** (842.92)		33887.48*** (10072.59)
Tiresomeness		-13903.97*** (2048.14)		-12972.73*** (2058.58)
Fear		10671.75*** (2461.51)		8183.73*** (2518.58)
Disgusting		11710.44*** (1865.25)		11495.95*** (1889.75)
Respect		10197.92*** (2077.03)		7898.73*** (2110.33)
Pathetic		8367.72*** (1609.85)		8003.56*** (1630.63)
Shame		-35623.54*** (10804.61)		-31326.58** (10858.77)
Recognize		-7668.00*** (2001.72)		-9001.16*** (2086.35)
*Attitude Categories*:				
Anti-Immigrant Attitude			3687.26** (1400.24)	3338.20* (1362.61)
Appeal to Emotion			456.81** (151.88)	426.38** (156.39)
Appeal to Authority			334.31*** (72.86)	363.65*** (74.15)
Family Orientation			90.61*** (19.58)	95.35*** (21.60)
*Controls*:				
Url Existence				2300.34** (742.12)
Content Length				.02 (.15)
Constant	3550.01*** (195.22)	257.76 (538.26)	3993.97*** (185.35)	-173.96 (546.90)
N	45,766	45,703	45,766	45,703

Note

***p < .001

**p < .01

*p < .05 (two-tailed).

Firstly, results in Model 1 show that petitions that fall into a mix of post-materialist (companion animals, human rights & gender equality, and political reform) and materialist (safety & environment) issues are more likely to receive signatures than others. Among sentiment categories, Model 2 suggests that petitions expressing negative emotions in face of wrongdoing such as (1) guilt, (2) seriousness, (3) fear, (4) disgusting, and (5) pathetic are related to receiving higher number of signatures, while petitions that are related to sentiment categories such as (1) tiresome, (2) shame, and (3) recognize are less likely to earn public support than others. Interestingly, petitions that involve sentiments such as showing respect to higher authorities are positively related to receiving great numbers of signatures from the public. In addition, results in Model 3 show that attitude categories such as anti-immigrant attitude and appeal to emotion as well as Confucian attitudes such as appeal to authority, and family orientation are positively and significantly associated with petitions receiving more signatures than others. Finally, the saturated model in Model 4 provide additional support for the findings in Models 1, 2, and 3.

Moving to the content analysis, we examine the keywords that frequently appear in those petitions that meet the threshold. Fig 2 visualizes those words by their frequency, first in a graph and second, in a word cloud.

**Fig 3:**
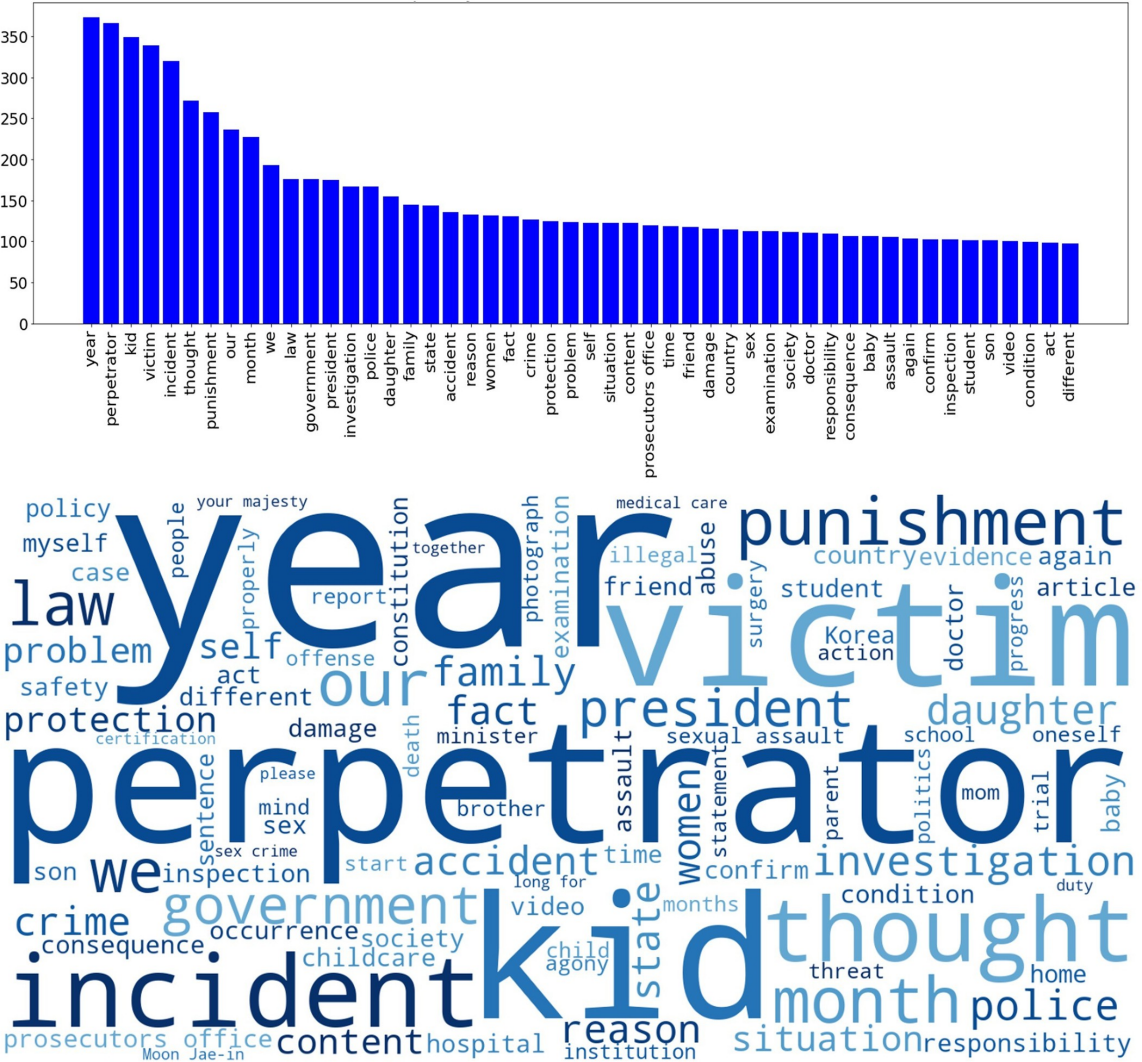
The number of petitions filed and passed by subject.

The graph in Fig 3 shows that the five keywords that appear most frequently are ‘year,’ ‘perpetrator,’ ‘kid,’ ‘victim,’ and ‘incident.’ In addition to the graph, the word cloud depicts the importance of each keyword by font size. While the term ‘year’ simply identifies the timing when an incident happened, the other four terminologies are linked to moral claims where *a kid becomes a victim of a perpetrator in an incident of violence*. In those petitions, the petitioner usually demands ‘punishment’ of the perpetrator by ‘law’ and further ‘investigation’ by the ‘government,’ the ‘police,’ or the ‘president.’ For example, in the petition that received the highest number of signatures from the public (2,715,626 signatures), the petitioner asked the government to reveal the identity of the criminal suspects who were involved in the ‘Nth Room’ case, a series of atrocious digital sex crimes on Telegram.

Next, we investigate the keywords that appear frequently in petitions that gained a great deal of public support by their subject categories in Table 3. Using the five categories that turn out to be statistically significant from stepwise regression models, we extract ten keywords that appear the most frequently in each of the five subjects, including companion animals, culture, arts, sports, and media, human rights and gender equality, political reform, and safety and environment.

**Table 3:** Keyword frequency in online petitions that meet the signature threshold in five subject categories.

	Companion Animals	Culture, Arts, Sports, & Media	Human Rights & Gender Equality	Political Reform	Safety & Environment
1	‘cat’	‘news’	‘victim’	‘prosecutors office’	‘perpetrator’
2	‘animal’	‘year’	‘perpetrator’	‘president’	‘family’
3	‘abuse’	‘broadcast’	‘incident’	‘people’	‘accident’
4	‘protection’	‘fact’	‘year’	‘your majesty’	‘our’
5	‘punishment’	‘press’	‘women’	‘Constitution’	‘punishment’
6	‘year’	‘research’	‘punishment’	‘minister’	‘year’
7	‘edible’	‘fake’	‘daughter’	‘government’	‘child’
8	‘slaughter’	‘channel’	‘thought’	‘reform’	‘us’
9	‘video’	‘approval’	‘investigation’	‘investigation’	‘month’
10	‘crime’	‘thought’	‘police’	‘country’	‘incident’

Different themes are revealed in the subjects of culture, arts, sports, and media and political reform. Based on the keywords that appear in the cultural category, we suspect that specific ‘news’ in a particular ‘year’ is usually ‘broadcasted’ by the ‘press’ and shared through these online petitions. Finally, in the subject of political reform, the petitioner—on behalf of the ‘people’—makes a request to the ‘prosecutors’ office,’ the ‘president,’ the ‘minister,’ or the ‘government’ to create reform against an institution, and Confucian attitudes are revealed in expressions such as ‘your majesty’ to propose political reform to the ‘president.’

All in all, our regression and content analyses reveal the characteristics of petitions that successfully attract public support. The results suggest that, between the five-year period when online petitions were sent to the administrative office, petitions that fall into post-materialist topics, such as human rights and gender equality, political reform, and companion animals, petitions that incite moral emotions or appeal to Confucian attitudes, and petitions that demand the apprehension of perpetrators on behalf of victims, are most likely to reach the signature threshold and to garner public support than others.

## Conclusion

Online platforms have been regarded as a fertile soil for citizens to freely express and share their opinions [17,38,39]. Online communication has been identified as an important vehicle for sharing and spreading grievances [16,40,41], and despite its limitations, national online petitioning has become one of the conventional and institutionalized ways of engaging in politics in contemporary democracies [3,4,13,22,23].

In this research, we focused on online petitioning in South Korea, a non-Western country with a consolidating democracy and a vibrant civil society. Our analysis identified how grievances are being shared by the public and which kinds of grievances garner support from the public in the online petitioning platform. Our results reveal that the major features of petitions that cross the signature threshold include appeals to a mix of post-materialist and materialist values, moral emotions, and Confucian attitudes, as well as moral claims asking for the apprehension of perpetrators on behalf of victims. The coexistence of modern and traditional values turns out to be a unique feature in a non-Western country such as South Korea.

Our results provide implications for understanding both the potentials and limitations of online petitioning in promoting democracy. First of all, the contents that online petitions contain can reflect the preeminent interests of the public. As our case study illuminates, the prevalence of post-materialist values, in addition to materialist, traditional, and Confucianist attitudes, becomes salient in what people want to share through online petitioning [34,35,37,42]. Subjects such as human rights, gender equality, and animal rights have increasingly driven attention from the public, and petitions that are related to these issues turn out to garner a great deal of support from the audience.

In addition, our analyses suggest that moral emotions play a critical role in drawing public support [26,27]. The dichotomy between victims and perpetrators has been employed widely to influence people’s morality, and these moral claims have been successfully utilized for online petitions to receive public support. It is noteworthy that moral emotions can serve as a double-edged sword in our contemporary society. The thematic collision between victims and offenders has been the foundation of increasing political polarization in contemporary democracy, and in some instances, it can obviously oversimplify the rather complicated reality. But in cases of crime or violence, where perpetrators and victims are more easily identifiable, moral emotions can translate into public pressure on the government to make proper changes.

At the same time, however, online petitioning platforms have limitations in bringing about radical and fundamental changes in the social structure. Since petitions usually focus on reporting and sharing a particular incident rather than asking for institutional reform, these platforms can simply serve as an individualized way of dealing with a social problem. In a related vein, the tendency of online petitions to reveal Confucian attitudes or primarily appeal to emotions can limit the potential of online petitioning systems to make social structural changes.

In addition, the digital divide inherent in online petition-signing may lead to problems of overrepresentation of a particular demographic group. Scholars have long been concerned about the effect of inequality in Internet usage between social classes and age groups [43–45]. Since social groups with higher incomes and levels of education, as well as younger populations, are more likely to initiate online petitions and show their support in online petitioning systems, the increasing visibility and popularity of these online platforms can enhance the inequality of political representation between social groups. Although the online platform that we focus on in this study do not keep information on the users who anonymously file petitions and write signatories, future studies may further examine the inequality of political representation in democratic regimes based on comprehensive data of users’ socio-demographic backgrounds and online activities.

Online petitioning platforms have spread to various governments, and the scholarship on democracy and social movements has begun to examine the potentials, as well as the limitations, of this new institutional form of political participation. Our study on the case of South Korea paves the way to further our understanding of how grievances receive public support in these nascent petitioning platforms in contemporary democracies.
